# Nurses’ intention to work after retirement, work ability and perceptions after retirement: a scoping review

**DOI:** 10.11604/pamj.2019.33.217.17568

**Published:** 2019-07-17

**Authors:** Wonpen Kaewpan, Karl Peltzer

**Affiliations:** 1 Department of Public Health Nursing, Faculty of Public Health, Mahidol University, Salaya, Phutthamonthon, NakhonPathom 73170, Thailand; 2 Department of Research and Innovation, University of Limpopo, Turfloop 0527, South Africa

**Keywords:** Work, post-retirement, intention, work ability, older nurses

## Abstract

Nurses have been identified as active workforce post-retirement. Little is known about nurses' intention to work post-retirement and their work ability and perceptions post-retirement. The aim of this scoping review is to identify studies that have investigated nurses' intention and recruitment strategies to work post-retirement and their work ability and perceptions post-retirement. PubMed, CINAHL, Web of Science, Nursing and Health Database and in addition Google scholar were searched using different keywords (retired nurses, retired RNs, work intention, work ability, work perceptions, and older nurses) and an independent reviewer cross-validated all the identified articles. Of the thirty-seven studies identified from the search, 26 papers were excluded based on exclusion criteria, with a total of 11 studies finally included in the study sample. The review found in two studies low and high intentions to work as nurses after retirement. Factors influencing both intentions and recruitment strategies to work as a nurse post-retirement focused on lighter and flexible work conditions, supportive environment, financial incentives and formal rehiring policies. Nurses working post-retirement reported benefits (intrinsic factors such as self-worth, overcoming social isolation) and barriers (such as job demands and reduced physical work ability). The review found low and high intentions to work as nurses after retirement and identified factors influencing both intentions and recruitment strategies to work as a nurse post-retirement and benefits and barriers of working as nurses post-retirement that can inform strategies to retain nurses post-retirement.

## Introduction

Globally, there appears to be a growing shortage of nurses [1-3]. In addition, the average age of the nursing workforce has been increasing [4, 5]. The retirement age for nurses differs by country, with e.g. no statutory retirement age in Australia [4]. Various statements have indicated the need of nurses to work as nurses in post-retirement, e.g. “flexible working after retirement can help older workers adjust” [6]. “Reactivation of retired nurses: a part time employment and understanding attitude of the work environment” [7]. “Reject retirement age of 65” [8]. “Bring nurses out of retirement to help ailing hospitals” [9]. For example in Thailand, the crisis of the nursing shortage is not only the failure to retain qualified and experienced nurses but also inadequate production. The research on policy on demand and supply projection of nurses for the period of 2017 to 2021 found that the shortage of skilled and experienced nurses in Thailand will become more severe in next 10 years due to early leaving of young nurses and retirement of senior nurses [10]. The results also revealed that improving policies in recruiting and retaining qualified nurses, creating positive practice environment and engagement, strengthening collaboration and resource sharing among all stakeholders are needed [10]. Moreover, maximizing the efficient use of nurses, particularly among the seniors, was also suggested to reduce the degree of severity of this problem, in line with Thailand’s Twelfth National Economic and Social Development Plan (2017-2021) [11]. Yet, there is a scarcity of research on how retired professional nurses can help in filling some of the gaps of the nursing shortage [12]. There is also little professional literature on the intention and ability of nurses to work as nurses post-retirement [12]. Some research seem to suggest that retired nurses are entering the workforce again because of financial reasons, which could have positive impact on the nursing shortage [12]. Previous reviews of research studies focused on the intention and work ability of nurses to retain them into employment until retirement [13-19], but we are not aware of a review of studies focusing on the intention, recruitment strategies, work ability and perception of nurses post-retirement, which prompted this scoping review.

## Methods

**Search strategies:** according to CRD guidelines on EQUATOR, a comprehensive search strategy was used to identify relevant studies. PubMed, CINAHL, Web of Science, Nursing and Health Database and in addition Google scholar were searched for combinations of keywords, such as retired nurses, retired RNs, work intention, work ability, work perceptions, and older nurses (Table 1). Specific inclusion and exclusion criteria were used to select relevant studies and an independent reviewer cross validated all the identified studies.

**Table 1:** Detailed search strategy

Database	Search Strategy	Results	Date
**PubMed**	(nurses work intention retirement) OR retired nurses work) OR retired RNs work	254	30 Sept 2018
**CINAHL Complete (EBSCOHOST)**	retired nurses work OR retired RNs work OR nurses work intention retirement	104	30 Sept 2018
**Web of Science**	TS=(nurses AND work AND intention AND retirement) OR TS=(retired AND nurses AND work) OR TS=(retired AND RNs AND work) Indexes=SCI-EXPANDED, SSCI, A&HCI, ESCI Timespan=All years	146	30 Sept 2018
**Nursing & Health Database**			30 Sept 2018
**S1**	mesh(nurses) AND work AND intention AND retirement	61	
**S2**	"retired nurses" AND work	218	
**S3**	“retired RNs” AND work	10	
**Google Scholar**			30 Sept 2018
**S1**	(nurses OR RNs) AND work AND intention AND post-retirement	1155	
**S2**	("retired nurses" OR “retired RNs”) AND work	1771	

**Inclusion criteria:** this scoping review included studies conducted between 2000 and 2018 that: (a) were published or electronically available in the English or Thai language; (b) were based on empirical quantitative and qualitative studies; (c) measured the intention and recruitment strategies to work as nurse post-retirement or beyond 65 years; and (d) focused on nurses working post-retirement or beyond 65 years in various settings and capacity.

**Exclusion criteria:** this scoping review excluded papers that: (a) involved insufficient details on either intention or working as a nurse post-retirement (not early but regular retirement); (b) included samples consisting of other health professionals (doctors, social workers); (c) described retirement experiences of nurses in general but not in the nursing profession; (d) retired nurses describing past nursing issues, (e) merely gave descriptive accounts of projects and/or programmes on the role of retired nurses without evaluation. The selected studies included in this scoping review were analysed based on their findings and reported in terms of the description of the intention of working as nurses post-retirement and work ability and perceptions of retired nurses.

## Current status of knowledge

**Figure 1:**
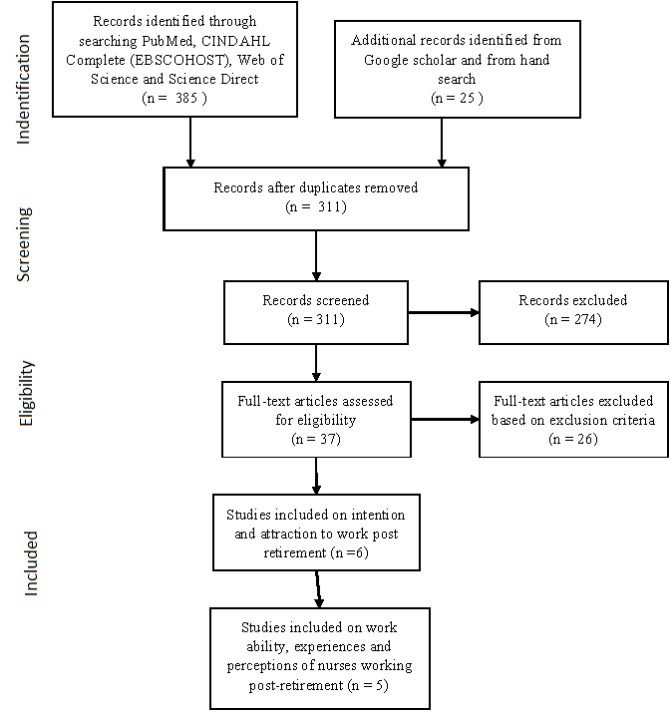
PRISMA 2009 flow diagramme showing study selection and data extraction process at every stage of the review

Using the described search strategy, a total of 37 studies that met the inclusion criteria were electronically identified from PubMed, CINAHL, Web of Science, Nursing and Health Database and in addition Google scholar and hand search. However, following application of the exclusion criteria, 26 of the 37 studies were excluded leaving 11 relevant studies. Four studies (3 quantitative and 1 qualitative) dealt with nurses' intention to work post-retirement, two qualitative studies on strategies and attitudes to attract nurses working post-retirement, and five studies (2 mixed method and 3 qualitative) on work ability and perceptions of working as a nurse post-retirement. This is illustrated below (Figure 1). Of these 11 identified studies, majority (n=9) were from high-income countries (5 from USA, 3 Australia and 1 Singapore) and 2 from middle-income countries (South Africa and Thailand).

**Nurses' intention to work post-retirement:** the review identified four studies on nurses' intention to work post-retirement [20-23]. One study in Australia reported a high intention to work as nurses post-retirement (73.2%) [20], while in Singapore only 18.3% wanted to work post-retirement (>65 years) [21]. Facilitators of the intention to work as nurses post-retirement included no shift work, reduced workload, job sharing or job rotation, short-term placements, financial incentives, supportive work environment, having a degree, disagreeing with attitudes not to work post-retirement [20-22]. Barriers to the intention to work as nurses post-retirement included work place incivility, remoteness of nurse practice settings, only informal system of post-retirement working arrangements [22] (Table 2).

**Table 2:** Method, sample and findings of identified studies

Method	Sample	Findings on nurses’ intention to work post**-**retirement
Quantitative (questionnaire) [20]	207 nurses and midwives working for the Department of Health (40-69 years old), Northern Australia	73.2%considered working post-retirement; Preferred types of engagement: reduced work load, job-sharing or job rotation, short-term placements, mentoring, research and policy development. Facilitators of post-retirement engagement among those who considered post-retirement: 93.2% financial incentives; 91.2%support from line management; 88.9%work environment support, 78.4%recognition of years of service, reduction in physical work load.
Quantitative (questionnaire) [21]	355 nurses in hospitals and primary care, 50 years and above, Singapore	18.3% wanted to work post-retirement (>65 years); Factors on wanting to work until age 65 and beyond (post-retirement): Nurses with a degree; Office hours (no shift work); Disagreed with wanting to stop working before age 65 when souse or close friend stops working; Disagreed with common attitude in society to stop working before 65 years
Qualitative [22]	6 Nurses in hospitals and community health, 50 years and older, and 9 of their managers, in Northern Australia	Potential for post-retirement engagement: flexible and part-time engagement, skill refresher course, financial incentives Barriers to post-retirement engagement: focus on younger Australian and overseas-trained nurses, remoteness of nurse practice settings, only informal system of post-retirement working arrangements
Quantitative [23]	384 nurses 50 years and older, USA	Job-related psychosocial factors associated with working post-retirement include lowering workplace incivility, providing generativity opportunities, encouraging relational job crafting, and cultivating work meaningfulness.
Method	Sample	Findings on strategies and attitudes to attract nurses working post-retirement
Qualitative [24]	21 nurse managers, South Africa	In relation to the effective use of retired nurses to alleviate nursing shortages, some nurse managers were in favour of their return, as they could apply their huge experiences effectively, while other nurse managers felt they would lack of productivity, as some of their experience may no longer be relevant or current. However, generally nurse managers agreed that retired nurses could play a role, in particular by making some changes to their workplace, such as given lighter duties and employing their skills in mentoring.
Focus groups [25]	7 focus groups, recently retired nurses, USA	Retired nurses can be rehired by using the following strategies to attract them: “Attractive rehiring policies with no loss of seniority; Part-time work opportunities/flexible schedules; Preferential scheduling accommodations; Financial incentives, including rehiring bonuses, salary adjustments for experience, increase matching contributions for older workers’ 401(k)or 403(b)plans; Health care coverage for retirees (i.e. Medigap coverage, prescription drug coverage).”
Method	Sample	Findings on work ability and perceptions of working as a nurse post-retirement
Quantitative (questionnaire)and qualitative (interview) [26]	147 retired nursing lecturers, age 60-79 years, Thailand	-Full-time employed as teaching staff (93.2%)and part-time employed as teaching staff (6.8%) -Positive view on post-retirement work conditions -Aware of ability to contribute to nursing school and profession -Overall were in good physical and mental health relative to declines of ageing (physically and congenital diseases)
Qualitative (in-depth interviews) [27]	6 retired older home health nurses, age 49-74 years, USA	Satisfaction about work (retired nurses): Patients and families (100%); positive intrinsic factors (83%); increased enjoyment (83%); Social relationships (co-workers and physicians) (83%) Dissatisfaction about work (retired nurses): Stressors (100%), work beyond work day (100%); pace of work (83%); technology (83%); specific patients/families (67%); other family responsibilities (67%)
Qualitative [28]	6 retired nurse mentors, aged 55-68 years, Australia	Benefits: “Enjoyed the capacity for engagement with new people, places and challenges.” Challenges: “Felt disconnected from the nursing and midwifery community postretirement; challenged to succeed in a new context of mentoring, one they did not experience themselves.”

**Strategies and attitudes to attract nurses working post-retirement:** the review identified two qualitative studies on strategies and attitudes to attract nurses to work post-retirement [24, 25]. Nurse managers in South Africa had mixed attitudes regarding nurses working post-retirement. Some argued that they could still have a useful role to play while others were against bringing retired nurses back [24]. Nurses in the US recorded different strategies to attract retired healthcare workers back into the work force, including attractive rehiring policies (financial, status, flexible work schedules) [25] (Table 2).

**Work ability and perceptions of working as a nurse post-retirement:** the review identified three studies on work ability and perceptions of working as a nurse post-retirement [26-28]. In all studies nurses in older homes, nurse mentors and nursing lecturers expressed benefits of working post-retirement (positive view, ability to contribute to nursing, satisfaction such as positive intrinsic factors, and enjoyed capacity for engagement with new people, etc.). Various challenges were also expressed in the form of overworking, handling technology, feeling disconnected from nursing community post-retirement, and their own physical decline due to ageing (Table 2).

**Work ability and perceptions of retired nurses working as volunteers:** the review identified two studies work ability and perceptions of retired nurses working as volunteers [29-30]. Retired nurse volunteers expressed several benefits of their work (enhanced self-worth, intellectual stimulation, opportunity to help others, and reduction of social isolation) and several challenges or barriers (such as physical problems, increased paperwork, new technology, lack of respect and licensing problems) (Table 2 suite).

**Table 2 suite:** Method, sample and findings of identified studies

Method	Sample	Findings work ability, experiences and perceptions of retired nurses working as volunteers
Mixed method, in-depth telephone survey [29]	23 retired nurse volunteers in clinics, 49-78 years, USA	Motivations: “Assist medically underserved persons (91%); give something back to community (91%); Always planned to volunteer in retirement (70%); want to continue or missed working (48%).” Deterrents to volunteering: “Physical problems (17%); discomfort with being out of practice so long (17%); not wanting to be tied down to a schedule (13%), notbeing aware of what they can do to help or where they can help (13%); the cost of malpractice/licensing (9%); and problemsgetting transportation to clinic (9%)”
Qualitative [30]	10 retired nurses volunteering as nurses and those who werenot volunteering, age 63-86 years, USA	Benefits: “Enhanced self-worth; intellectual stimulation; reduced social isolation, opportunities to help others.” Challenges and barriers: “Increased paperwork, new technology; difficulty finding nursing-specific volunteer opportunities; resistance from health care organizations; and a lack of respect for what these nurses know.”

This review identified 11 studies on nurses' intention to work after retirement and work ability and perceptions after retirement. The intentions to work after retirement ranged from 18.3% in Singapore [12] to 73.2% in Australia [11]. Facilitators of both intentions to work as a nurse post-retirement and strategies to attract nurses post-retirement were similar, including lighter and flexible work conditions, supportive environment and financial incentives [11-13]. Likewise, barriers of both intentions to work as a nurse post-retirement and strategies to attract nurses post-retirement were similar, including lack of attractive rehiring policies, lack of formal system of post-retirement working arrangements, un supportive environment and inflexible work schedules [4, 12-16] on the retention of older nurses (up to retirement) found similar facilitating and challenging factors, ranging from supportive environment, reduced and flexible workload, to improved education and training. Nurses working as nurse or nurse volunteer in post-retirement reported both beneficial and challenging aspects of their work. Benefits included intrinsic factors (self-worth, overcoming social isolation) and barriers included job demands and reduced physical work ability [26, 28-30]. Current retirement policies in the various countries may need to refocus by introducing more flexible opportunities for post-retirement employment of nurses [13]. Other programmes seem to successfully employ nurses post-retirement in the academic sector, such as in the study from Thailand [17] and Baldwin *et al*. [31] describe the integration of retired nurses into a new graduate orientation programme in the US.

**Limitations:** most studies (9 of 11) included in this scoping review had been conducted in high-income countries, lacking generalize ability to low- and middle- income countries. Due to the scarcity and descriptive nature of the data, no quality assessment of the specific studies was conducted.

**Funding:** the research was carried out within the framework of research project “development of intermediate and long term care model for dependent elderly by enhancing competency and employment of retired nurses as community nurse managers”, supported by Mahidol University, and was partially supported for publication by Faculty of Public Health, Mahidol University, Bangkok, Thailand.

## Conclusion

The scoping review found low and high intentions to work as nurses after retirement, and identified factors influencing both intentions and recruitment strategies to work as a nurse post-retirement and benefits and barriers of working as nurses post-retirement that can inform strategies to retain nurses post-retirement. Too few studies have been conducted on the topic emphasizing the need for more quantitative and qualitative research, especially in low- and middle- income countries.

### What is known about this topic

- Previous reviews of research studies focused on the intention and work ability of nurses to retain them into employment until retirement;
- We are not aware of a review of studies focusing on the intention, recruitment strategies, work ability and perception of nurses post-retirement.

### What this study adds

- The intentions of nurses to work after retirement ranged from 18.3% in Singapore to 73.2% in Australia;
- Nurses working as nurse or nurse volunteer in post-retirement reported both beneficial (self-worth, overcoming social isolation) and challenging (job demands and reduced physical work ability) aspects of their work;
- Current retirement policies in the various countries may need to refocus by introducing more flexible opportunities for post-retirement employment of nurses.

## Competing interests

The authors declare no competing interests.
